# A novel mechanism of ferroptosis surveillance offers promising therapeutic avenues for breast and prostate cancers

**DOI:** 10.1186/s43556-023-00140-4

**Published:** 2023-09-22

**Authors:** Rongyang Xu, Luyao Wang, Shanqiang Qu

**Affiliations:** 1 Department of Neurosurgery, Nanfang Hospital, Southern Medical University, Guangzhou Dadao Bei Street 1838, Guangzhou, 510515 Guangdong China; 2 The Laboratory for Precision Neurosurgery, Nanfang Hospital, Southern Medical University, Guangzhou, 510515 Guangdong China; 3 The First Clinical Medical College of Southern Medical University, Guangzhou, 510515 Guangdong China

A recent study entitled “Ferroptosis Surveillance Independent of GPX4(Glutathione Peroxidase 4) and Differentially Regulated by Sex Hormones” has uncovered a novel regulatory mechanism for ferroptosis. This study highlights a novel pathway for monitoring ferroptosis that is independent of GPX4 regulation and is instead differentially regulated by sex hormones. Published in *Cell* on May 23, 2023 [1], this paper demonstrates that Membrane Bound O-Acyltransferase Domain Containing 1 (MBOAT1) and Membrane Bound O-Acyltransferase Domain Containing 2 (MBOAT2) can serve as novel targets for cancer treatment, offering new possibilities for treating cancers with specific genetic backgrounds (Fig. 1).

**Fig. 1:**
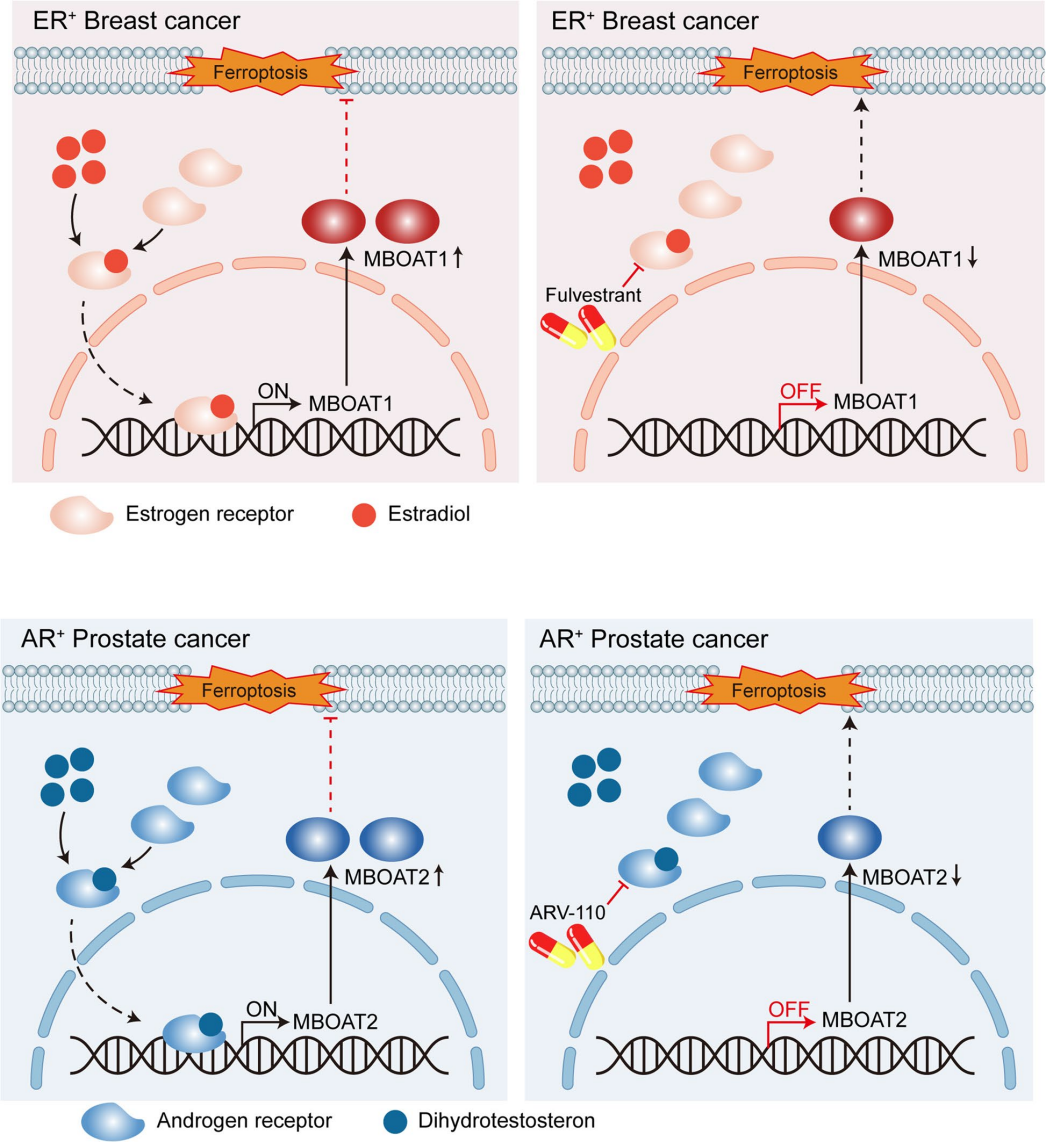
A Novel Mechanism of Ferroptosis Surveillance for Breast and Prostate Cancers. MBOAT1 and MBOAT2 are regulators of ferroptosis, whose action is tied to sex hormones and has therapeutic implications for estrogen receptor-positive breast cancer and androgen receptor-positive prostate cancer, respectively. MBOAT1 and MBOAT2 independently suppress ferroptosis through phospholipid remodeling without dependence on GPX4

Ferroptosis is a type of cell death caused by the accumulation of polyunsaturated fatty acids in cell membranes, which represents a common form of cell death [2]. Ferrous ions play a crucial role in ferroptosis by initiating lipid peroxidation, and amplifying the accumulation of lipid peroxides. Ferroptosis has been found to play a vital role in cancer suppression. There are currently two main monitoring and regulatory mechanisms for ferroptosis in cancer [3]. The first mechanism is the glutathione (GSH)/glutathione Peroxidase 4 (GPX4) axis. GPX4 is the only mammalian enzyme known to reduce phospholipid hydroperoxides (PLOOH) to the corresponding phospholipid alcohols. Another regulatory mechanism involves the production of antioxidant cell metabolites through enzymes such as ferroptosis suppressor protein 1 (FSP1), dihydroorotate dehydrogenase (DHODH), Nitric Oxide Synthase 2 (NOS2), and GTP Cyclohydrolase 1 (GCH1) [2, 4]. These metabolites have the unique ability to trap free radicals, effectively terminating the phospholipid peroxidation process to prevent ferroptosis. However, in certain cases, the ferroptosis regulators GPX4 and FSP1 may be defective. Thus, it is imperative to identify new regulatory mechanisms and targets for ferroptosis in cancer.

MBOAT1 and MBOAT2 are newly discovered endoplasmic reticulum (ER) membrane proteins that function as L-type acetyltransferases. While earlier studies have identified their role in various cellular processes, their specific involvement in ferroptosis regulation remains unclear and requires further investigation. The study utilized a genome-wide CRISPR activation screening approach to identify the role of MBOAT2 in ferroptosis. The results revealed a significant enrichment of MBOAT2’s guide RNA in viable cells under ferroptosis-induced conditions. Functionally, overexpression of MBOAT2 was shown to significantly inhibit ferroptosis induced by Ras-selective lethal 3 (RSL3) or glutathione depletion. Mechanistically, MBOAT2 significantly inhibited ferroptosis in human fibrosarcoma cells, even when both GPX4 and FSP1 were knocked out. These findings suggest that MBOAT2 can prevent ferroptosis through a phospholipid-remodeled mediated monitoring mechanism, that is independent of GPX4 and FSP1. Likewise, MBOAT1, another acyltransferase that inhibits lipid peroxidation-associated cell death, has been shown to rescue ferroptosis induced by GPX4 knockout or RSL3. Drawing from these observations, an intriguing hypothesis emerges: in biological processes subject to meticulous regulation by sex hormones, such as reproductive development, an upregulation of MBOAT1/2 might be imperative for suppressing ferroptosis in pertinent organs.

Subsequently, the researchers investigated the factors driving the upregulation of MBOAT1 and MBOAT2 in cancer. Interestingly, while MBOAT1 and MBOAT2 seemingly suppress ferroptosis through a shared biochemical pathway, their regulation by sex hormone signaling differs, implying distinct biological functionalities. MBOAT2 exhibited specific upregulation in prostate cancer and its expression was positively correlated with androgen receptor (AR) mRNA expression in prostate cancer, suggesting that MBOAT2 may be a target of AR in prostate cancer cells. Moreover, AR antagonists could increase the sensitivity of AR-positive prostate cancer cells to ferroptosis by downregulating MBOAT2 directly. MBOAT1 exhibits high expression in several female-related cancers, including ovarian, breast, and endometrial cancers, indicating that MBOAT1 may be regulated by estrogen receptor (ER) signaling. Interestingly, the study revealed that ER antagonists could enhance the sensitivity of ER-positive breast cancer cells to ferroptosis by downregulating MBOAT1. While the present study demonstrates the inhibitory effects of MBOAT1/2 on ferroptosis, a comprehensive examination of all LPLATs and their modulation by cancer signaling mechanisms is yet to be undertaken.

In conclusion, the study reveals that MBOAT1 and MBOAT2 can inhibit ferroptosis through phospholipid remodeling independently of GPX4. The research also suggests that AR and ER transcriptionally upregulate the expression of MBOAT1 and MBOAT2, respectively, and inhibition of these receptors downregulates their transcriptional expression, sensitizing prostate cancer and breast cancer cells to ferroptosis. Collectively, these findings highlight MBOAT1 and MBOAT2 as promising targets for prostate and breast cancers. This study raises several questions about the presence of the novel monitoring mechanism of ferroptosis in other solid cancers. Future experiments could provide insights into the specific downstream mechanisms of the MBOAT1 and MBOAT2 proteins.

## Data Availability

The datasets generated during and/or analysed during the current study are available from the corresponding author on reasonable request.
